# Professionals’ adaptive expertise and adaptive performance in educational and workplace settings: an overview of reviews

**DOI:** 10.1007/s10459-022-10190-y

**Published:** 2022-12-12

**Authors:** Els Pelgrim, Elske Hissink, Lotte Bus, Marieke van der Schaaf, Loek Nieuwenhuis, Jan van Tartwijk, Wietske Kuijer-Siebelink

**Affiliations:** 1 Learning and Innovation Centre, Avans University of Applied Sciences, Breda, The Netherlands; 2 Department of Research on Learning and Education, Radboud University Medical Center, Radboudumc Health Academy, Philips van Leydenlaan 25, 6525 EX NIJMEGEN Nijmegen, The Netherlands; 3 School of Education, HAN University of Applied Sciences, Nijmegen, The Netherlands; 4 Utrecht Center for Research and Development of Health Professions Education, University Medical Center Utrecht, Utrecht, The Netherlands; 5 Faculty of Social and Behavioural Sciences, Utrecht University, Utrecht, The Netherlands

**Keywords:** Adaptive expertise, Adaptive performance, Learning of professionals, Overview of reviews

## Abstract

Professionals will increasingly be confronted with new insights and changes. This raises questions as to what kind of expertise professionals need, and how development of this expertise can be influenced within the contexts of both education and work. The terms adaptive expertise and adaptive performance are well-known concepts in the domains of education and Human Resource Development respectively. The literature, however, lacks a conceptual overview. Our research seeks to provide an overview on how adaptive expertise and adaptive performance are conceptualized. In addition we looked for what individual, task and organizational characteristics relate to adaptive expertise. We mined information drawn from existing reviews in an overview of reviews. Nine reviews met the inclusion criteria. Adaptive performance is best referred to as the visible expression of an adaptive expert and this is triggered by ‘change’. The scope of this ‘change’ lies somewhere between change that is ‘new for the learner’ and change that is ‘new for everyone in the whole world’. The extent to and way in which a learner or professional is able to deal with this change depends on the maturity of the learner or professional. We found numerous individual, task and environmental characteristics related to adaptive expertise and adaptive performance. The nature and relation of these characteristics, and their specificity in relation to adaptive expertise and adaptive performance are visualized in a figure, but also provide several suggestions for future research.

## Introduction

Working in an era of increasing complexity demands that professionals are trained to have the ability to apply their extensive knowledge base, create new knowledge (Mylopoulos et al., 2018a) and rapidly acquire new skills as needed. This is seen in key professions that shape future society, including industry, government, the military, and healthcare, and is needed in every domain and context where learning is central to daily activities (Ward et al., 2018a). In most domains, professionals will be frequently confronted with new insights and context changes, and professionals that respond well to these changes are referred to as “adaptive experts” in the literature (van Tartwijk et al., 2023).

So far, there is a variety in conceptualization and use of adaptive expertise and there is a sense of urgency for clarification. For instance, in the educational context, the theories and models that generally underpin educational programmes do not account for the adaptive expertise needed in, for example, the changing health care context (Mylopoulos et al., 2018a). Contrarily, in work contexts, knowledge about individuals’ adaptability is more and more used to understand and estimate how employees respond to changing and new work situations (Van Dam & Meulders, 2021) and to contribute to developing and training employees for a specific role (Oprins et al., 2018).

To further stimulate the development of future professionals in educational environments and in the work context, insight is needed in what kind of expertise professionals need and how this expertise can be influenced within both the education and work contexts.

Experts are often defined as individuals who perform tasks representative for their domain more effectively and more efficiently than others (van Tartwijk et al., 2023). Traditionally, research into expertise development is carried out on routine tasks for which differences in performance between those performing at a novice or expert level of proficiency can be rather easily detected (Ward et al., 2018a). Besides, researchers have been interested in the savings to be made from engaging in prior learning when re-learning the same task, and the degree of transfer of previous learning to a similar task (Ward et al., 2018a). Using this definition of expertise, which links expertise to the performance of tasks within a domain, renders studying expertise in the field of emergent professions difficult. Here, performance is not about re-learning the same task and therefore cannot easily be simulated or reduced to single-task performance (Ward et al., 2018a). In addition, more thinking is needed on how to develop expertise among professionals facing complex societal issues, or on educating experts for new or rapidly growing occupational fields (Nieuwenhuis et al., 2022).

In educational environments, the start of this rationale lies in the work of Hatano and Inagaki (1986). They provided a new perspective on the expertise needed for emergent professions and on how this could be developed. They discern *routine expertise* and *adaptive expertise.* Hatano and Inagaki conceptualized these terms as the poles of one dimension, whereby routine expertise is the execution of high-quality procedures in order to act efficiently and accurately, while adaptive expertise is the power to develop new solutions to professional problems or even the power to develop new problem-solving methods. Hatano and Inagaki (1986) suggest three factors that enhance the development of adaptive expertise: (1) a random context that forces professionals to adapt their skills, based on careful observation and interaction; (2) a safe environment where rewards do not depend on performance; (3) a working context which values quality over efficiency. In line with this, other authors (for example, Mylopoulos et al., 2018b; van Tartwijk et al., 2023) have formulated ways in which education can be provided to support the development of adaptive expertise, however most of these recommendations have not been examined empirically.

In the Human Resource Development (HRD) research field, there was a need for a term for the complexity of performance, congruent with the emergence of adaptive expertise in the field of expertise development earlier. In 1999, Hesketh and Neal (1999) introduced the term *adaptive performance*. This term was first defined (Hesketh & Neal, 1999) as employees’ capability to adapt to rapidly changing work situations. Before the term adaptive performance was used, employee performance was most frequently described using criteria such as productivity, sales, or the quality of goods and services (Charbonnier-Voirin, 2012). Defined in this manner, the focus is solely on outcomes. In line with the conclusions in traditional expertise research, the conceptualization of performance in terms of outcomes do not represent complex work situations and is therefore less useful. Consequently, new models including the notion of adaptive performance were developed (Charbonnier-Voirin, 2012).

Today, we see variation in conceptualization of adaptive expertise and adaptive performance for educational contexts and work contexts. In recent literature reviews (for example, Bohle Carbonell et al., 2014; Kua et al., 2021), adaptive expertise and adaptive performance are regularly considered as interrelated or even interchangeable concepts. The authors of these reviews did not distinguish results from publications on either adaptive expertise or adaptive performance. Besides literature in which the two concepts are used interchangeably, research exists that focusses specifically on one of the two concepts. In the Oxford Handbook of Expertise for example, Bohle Carbonell and Van Merriënboer carried out a co-citation analysis for key concepts around adaptive expertise (Bohle Carbonell & van Merriënboer, 2018). They excluded adaptive performance from their analysis as they adopted a solely educational viewpoint for their co-citation analysis, excluding the HRD knowledge domain. They conclude that individuals with adaptive expertise have information not only about how to apply a skill, but also know when and why to apply that skill. The notion is that adaptive experts explicitly seek to learn from their experiences. The aim of adaptive experts is not only to complete a task, by applying knowledge and skills, but also to develop their knowledge and skills (Bohle Carbonell & van Merriënboer, 2018). These components, which are deemed essential for adaptive expertise, are not automatically visible in performance.

Variety in conceptualization of the concept of adaptive expertise is also visible between different authors within the Oxford Handbook of Expertise. In contrast to the chapter by Bohle Carbonell and Van Merriënboer that deals explicitly with adaptive expertise (Bohle Carbonell & van Merriënboer, 2018), Ward and colleagues argue, in the reflection section of the Handbook, that a separate designation for adaptive expertise is not necessary (Ward et al., 2018b). They point out that experts, by nature of their expertise, have developed specific characteristics that allow them to be more adaptive than non-experts. Ward et al. (2018b) state that both conceptual understanding and flexible decision-making are two parts of an integrated dynamic system that give rise to successful adaptation in both familiar and new complex contexts within one’s domain of expertise. While they recognize adaptivity in the expert, sometimes referred to as flexibility, they conclude that this is inextricably linked to expertise, just as routine skills. In their opinion, this renders the term expertise satisfactory for the concept as a whole.

Lastly, there is no clarity as to the extent to which adaptive expertise or adaptive performance is domain specific. Hatano and Inagaki (1986) suggest that adaptive expertise is domain-specific because it is developed by accumulating experiences. Moreover, Ward and colleagues assert domain specificity (Ward et al., 2018b). On the other hand, other authors argue that adaptive expertise should be seen as tackling new tasks that could fall both within and outside the domain of the expert (Bohle Carbonell et al., 2014). Further, as yet there is no answer to the question as to how novel a situation must be in order to demonstrate adaptive expertise, and at what point a situation becomes so novel that even an individual with adaptive expertise cannot handle it (Bohle Carbonell et al., 2014).

All in all, the literature lacks conceptual clarity. We can discern different discourses where adaptive expertise or adaptive performance seem to be the key concepts. In addition, we see that there is insufficient connection between the knowledge bases underpinning these concepts in the education and HRD domain. The two concepts seem related, but research into these concepts has different roots. Our aim with this publication is to provide more insight into the conceptualization of these concepts by bringing together the knowledge bases around adaptive expertise and adaptive performance and thereby contribute to the development of professionals in emerging professions in an evidence-informed manner.

Our research aims to provide this clarity by scrutinising definitions, existing models and frameworks for both adaptive expertise and adaptive performance in educational settings and work contexts. In addition, we look for clues about characteristics that stimulate the development of adaptive expertise or adaptive performance.

We mined information drawn from existing reviews into an overview of reviews. This is an explicit and systematic method to search for and identify multiple reviews on related research questions in the same topic area for the purpose of extracting and analysing their results across important outcomes (Fernandes et al., 2022). Overviews are used to map the available evidence and identify gaps in the literature (Lunny et al., 2018). Our overview of reviews summarizes the evidence on adaptive expertise and adaptive performance in the field of expertise and professional development. The research questions are:

1. How are adaptive expertise and adaptive performance conceptualized?

- Which models or theoretical frameworks are used to describe adaptive expertise or adaptive performance?

2. What individual, task and organizational characteristics relate to adaptive expertise or adaptive performance?

## Methodology

### Search strategy

The literature search was conducted in April 2021, using two major scientific multidisciplinary databases: Scopus and Web of Science. These databases were chosen based on the access they provide to high quality research in the domains of Research and Development (R&D) and Education across different disciplinary fields. The timespan was limited to publications released between 1986 (emergence of the concept of adaptive expertise) and April 2021. The search strategy used was developed in an iterative process by EP, EH, MvdS, LN, JvT and WK in consultation with a research librarian. Table 1 shows the keywords used. Although our research questions focus on adaptive expertise and adaptive performance, we searched more broadly than specifically on those words to be able to include reviews using an umbrella term that may include underlying publications related to our core concepts. Additional material sets out the complete search strategies per database (see: Availability of data and materials). The selected publications were managed and filtered for duplicates in Endnote™ citation manager. Deduplicated publications were exported and analysed using Rayyan (Ouzzani et al., 2016).

**Table 1:** Keywords used in the search

Keywords used in the search
Feature 1 – terms related to ‘the person’^1^
expert* OR learner OR professional OR student OR resident* OR intern OR trainee OR novice OR worker OR employee OR scholar OR individu*
Feature 2 – terms related to adaptivity^1^
adaptiv* OR adapta* OR flexib*
Feature 3 – terms related to the result and/or development of adaptivity
develop* OR learn* OR perform*
Feature 4 – terms related to the document type^2^
review OR “meta analysis" OR “systematic map" OR overview OR synthesis

^1^ We used the ‘near 3’ option for features 1 and 2 so that only publications where these words were mentioned close together in the title or abstract were included

^2^ We also ran features 1, 2 and 3 in combination with the ‘review’ document type option in Web of Science and Scopus

### Screening and selection

EP, EH, LB, LN and WK conducted the screening process, using a two-step approach. During the first step, the titles and abstracts were screened using the following criteria: (a) the abstract is in English, German or Dutch; (b) it is a literature review; (c) the concept of study is adaptive expertise, adaptive performance or a concept with a similar conceptualization. Each publication was independently screened by two researchers. In the event of disagreement, a third researcher also independently screened the publication. If two out of the three researchers included the publication, it was retained for full-text screening. If two out of three excluded the publication, the three researchers came to a decision on whether to include or exclude it after discussion.

In the second phase, the full text of the publication was screened. We used the same inclusion criteria supplemented by: d) the publication answers at least one of our research questions; e) the method section of the publication clarifies how search and selection was executed and f) books or book chapters are published by a scientific publisher OR journal articles are published in an SSCI-journal.

### Data analysis

We extracted general information from the included publications, such as: year of publication, review type and core concepts examined. Furthermore, information regarding our research questions was extracted by EP, LB and WK. All publications were discussed by two out of these three researchers. The following information was extracted:

- conceptualization of the concept used in the publication;
- results with regards to models or theoretical frameworks;
- characteristics that are considered to be related to the concept:
  - individual characteristics;
  - (job) task and/or training characteristics;
  - characteristics of the learning or work environment.

### Positionality

The research project focuses on conceptualizing adaptive expertise and adaptive performance, following the research traditions of both the domain of educational sciences and the domain of HRD. During two-weekly meetings with the main researchers (EP, EH, LB and WK), monthly meetings with all members of the research team, and three meetings (in the beginning, middle and end) with stakeholders from the research consortium (representing eleven institutions of Higher Education in the Netherlands), assumptions and perspectives were questioned and the data collection, analysis, and interpretation were shaped.

To be able to analyse all different definitions, conceptualizations and descriptions identified, working in a multidisciplinary team which included various research orientations and different disciplinary backgrounds was deliberately chosen. The team’s expertise included educational science (EP, EH, LN), e.g. teacher education (JvT), health professions education (EP, EH, JvT, MvdS, WK) and industrial innovation education (LN). In the research team researchers bring in both psychological perspectives, e.g. creativity, interpersonal communication, and expert perception (JvT, MvdS) and sociological perspectives, e.g. change processes in organizations (LN, MvdS). The multi-disciplinarity of the research team and other consortium members was valuable during these discussions, as it provided an opportunity to triangulate knowledge and expertise from different professional backgrounds.

## Results

**Fig. 1:**
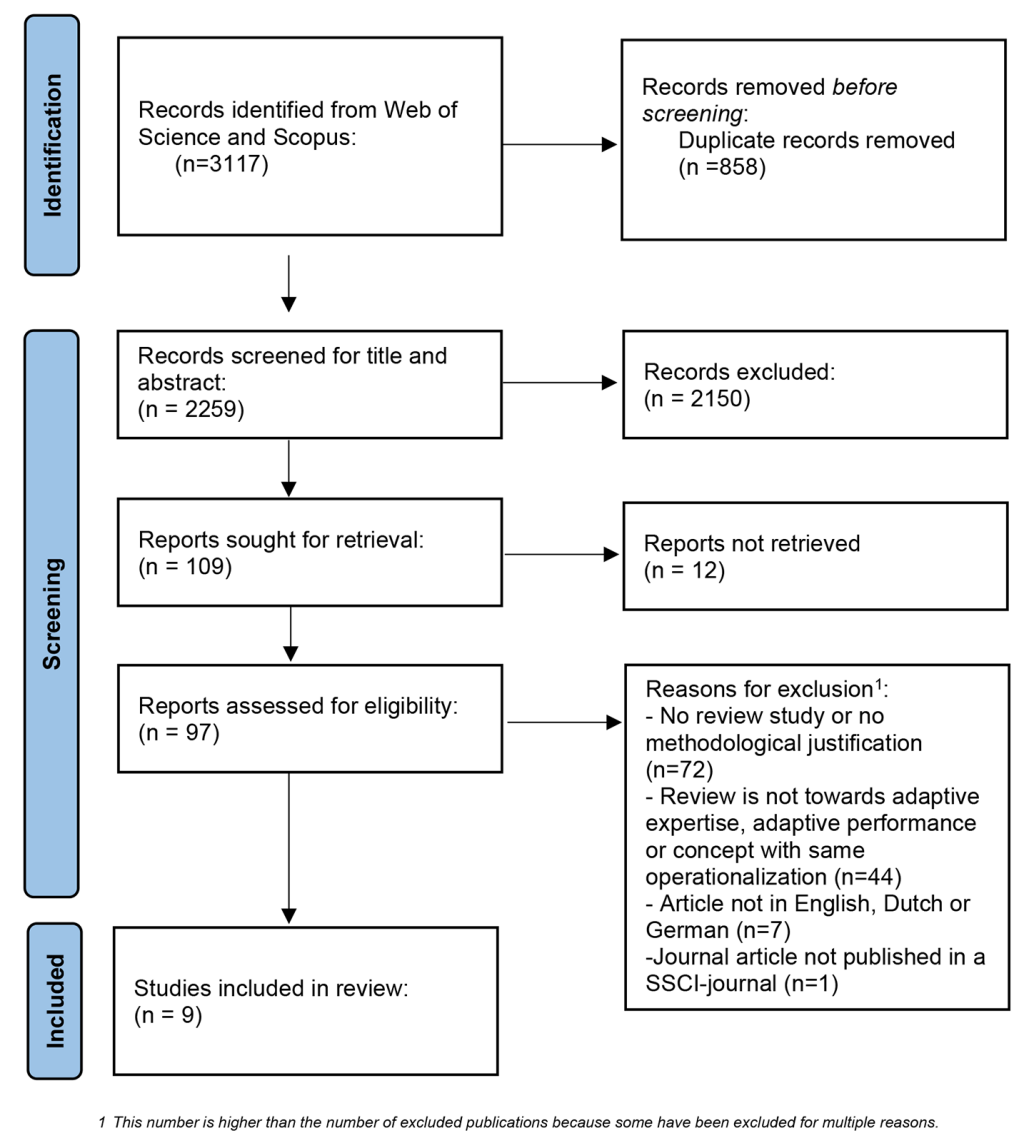
Publication PRISMA Flow Diagram (Moher, 2010)

In the results section we will set out the extracted information in three tables, as well as summarizing and putting it into context in the text below. In total, we identified 3,117 publications from the databases. After deduplication, the title and abstract of 2,259 records were screened. 109 publications were found to meet the inclusion criteria. However, 12 of the publications were not accessible. The remaining 97 publications were included for full-text screening. After this step, nine included publications were subjected to data analysis. See Fig. 1 for more details about the publication selection process and additional material (see: Availability of data and materials) for detailed information about reasons for exclusion per publication during full text screening. The nine publications selected were all journal articles published in English.

### Core concepts

The general information generated from the included publications, including the core concepts (and wordings used for these concepts) that each publication focusses on, is shown in Table 2.

**Table 2:** General information about included publications, sorted by publication year (ascending)

Reference	Review type^1^	Core concepts mentioned	Participants	Social context
Bohle Carbonell, K., Stalmeijer, R.E., Könings, K.D., Segers, M. & Merriënboer, van, J.J.G. (2014)	Systematic review	• Adaptive expertise • Adaptive expert • Adaptive performance	Employees, and students	Studies included from different contexts: workplace (10), simulation (5), training/education (6). Workplace studies were conducted in several industries (hospitality = 2; military = 1; aerospace = 1; electricity company = 1; and government agency = 1) and also across industries (4). The studies conducted in educational contexts dealt with experienced firefighters (2) and engineering pupils/students (4).
Baard, S.K., Rench, T.A. & Kozlowski, S.W.J. (2013)	Narrative review	• Performance adaptation	Employees	Individuals and teams in the context of the workplace.
Bartone, P.T., Krueger, G.P. & Bartone, J.V. (2018)	Systematic review	• Adaptability to isolated, confined and extreme environments	*Not mentioned. Most probably employees only.*	Isolated, confined, and extreme (ICE) environments. This includes environments and situations characterised by social isolation, confined, or restricted space and movement, persistent danger, and austere, harsh living conditions.
Ward, P., Gore, J., Hutton, R., Conway, G.E. & Hoffman, R.R. (2018c)	Critical interpretive research synthesis	• Expertise • Adaptive skill • Adaptive performance • Adaptive framing • Flexible execution (= adaptive planning or replanning) • Cognitive flexibility theory	*Not mentioned*	Not mentioned
Park & Park (2019)	Integrative literature review	• Adaptive performance • Adaptability • Adaptive ability • Adaptive expertise • Adaptivity	Employees	The various industries included manufacturing, banking, IT, hotel, healthcare, sales, the military, government and education.
Foster, C.J., Plant, K.L. & Stanton, N.A. (2019)	Systematic review using grounded theory approach	• Adaptation in safety management	*Not mentioned. Most probably employees only.*	Safety science. Safety of complex socio-technical systems.
Wallin, A., Nokelainen, P. & Mikkonen, S. (2019)	Integrative approach	• Development of expertise • Professional learning	• Higher education • Professional training • Professional doctorate • Postgraduate education • Further education • • Work-based higher education	Industry-university partnerships; higher level education (postgraduate, further education, doctoral education). Mostly published in multidisciplinary domains (8), the domains of medicine (3), teacher education (3) and business (2). Other domains (occupational risk management, police training and public works) were mentioned once.
Stasielowicz, L. (2020)	Meta-analysis	• Cognitive ability as a predictor of performance adaptation	Employees and students	No particular setting.
Kua, J., Lim, W.S., Teo, W. & Edwards, R.A. (2021)	Scoping review	• Adaptive expertise • Complex adaptive systems • Preparation for future learning (PFL) • Innovative problem solving • Innovativeness • Mental flexibility • Management of complexities • • Approach to novel situations	Learners or teachers of complexities and adaptive expertise	Including studies from many different disciplines within the context of education. Dichotomy identified between articles within health professions education (14) and non-health professions education (43) (engineering, special education, teaching, restaurant management and transport).

^1^ as reported in the publication

There seems to be little consensus on the terminology used to describe the concepts of adaptive expertise and adaptive performance, as different terminology is used in different publications. This impedes conceptual clarity of the concepts. We found that adaptability, adaptivity, adaptive expertise, adaptive skill, adaptive performance and performance adaptation are used in different publications, but without a clear conceptual difference. Some of the included publications focus specifically on the core concept itself, whereas other publications mainly focus on the predictors of adaptive expertise or adaptive performance.

**Fig. 2:**
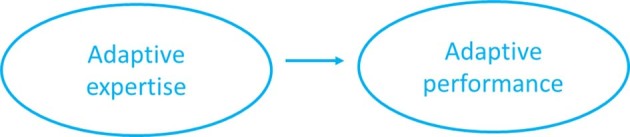
Theoretical representation of the results (core concepts)

The notion that adaptive performance is the visible behaviour resulting from adaptive expertise (Bohle Carbonell et al., 2014) or that individuals who are able to demonstrate adaptive performance should be identified as adaptive experts (Kua et al., 2021) is proposed by some authors, but not widespread. Also, the notion that adaptive skill is the logical condition for having expertise is found (Ward et al., 2018c), but not enfolded by all authors. The above shows that adaptive expertise and adaptive performance are not synonymous with each other, but related. In accordance with above, Fig. 2 shows the notion to view performance as the visual expression of expertise. The arrow in Fig. 2 does not imply a linear relationship, but having adaptive expertise is conditional on being able to perform adaptively. Adaptive expertise can be present without being visible in performance.

Of the nine included publications, two reviews focus explicitly on education (primary and secondary education, as well as higher education and postgraduate education from both learner and teacher perspectives), five reviews focus on a work context with different types of work in different domains, or combine the educational and work contexts, with different types of work in different domains, while two reviews do not explicitly mention the context (see Table 2). The publications that focus on the educational context usually use the term adaptive expertise. The publications that focus on other contexts are inclined to use the term adaptive performance or performance adaptation.

Based on Table 2 and the analysis of the wordings used to describe the core concepts in the different publications, we conclude that there is no congruent use of language in the field of adaptive expertise and adaptive performance research. Many different terms are used, and these terms seem to mean different things in different publications. For the readability of the results section, we will use the term adaptive expertise for all the terms used in the included publications, following the preliminary theoretical representation of the results shown in Fig. 2.

### Conceptualization

In line with our goal to provide clarity, we have listed and reviewed all the definitions, models and frameworks used in the included publications. An expanded version of this list is available (see: Availability of data and materials). Table 3 shows the different conceptualizations that are used in each of the publications.

**Table 3:** Conceptualizations used in the publications

Reference	Conceptualization used in the publication
Bohle Carbonell, K., Stalmeijer, R.E., Könings, K.D., Segers, M. & Merriënboer, van, J.J.G. (2014)	The ability to quickly get accustomed to change. This original conceptualization of adaptive expertise can be refined in the following point, addressed by the first two research questions: Adaptive expertise has similar, but not the same, basic components than routine expertise. They share the same extent of domain knowledge and skills, but differ in their knowledge representation. These representational differences have been shown to lie within the organization and abstraction of knowledge. New characteristics of adaptive expertise not mentioned previously are the importance of being confronted with novel situations and learning new tasks. Past experience related to dealing with other people and their viewpoints does not relate to adaptive expertise.
Baard, S.K., Rench, T.A. & Kozlowski, S.W.J. (2013)	They define performance adaptation as cognitive, affective, motivational and behavioural modifications made in response to the demands of a new or changing environment, or situational demands.
Bartone, P.T., Krueger, G.P. & Bartone, J.V. (2018)	The capacity to respond appropriately to changed or changing situations: the ability to modify or adjust one’s behaviour when encountering different circumstances or people. Further defining adaptability: the ability and willingness to anticipate the need for change, to prepare for that change, and to implement changes in a timely and effective manner in response to the surrounding environment.
Ward, P., Gore, J., Hutton, R., Conway, G.E. & Hoffman, R.R. (2018c)	Timely changes in understanding, plans, goals and methods in response to either an altered situation or updated assessment of the ability to meet new demands, that permit successful efforts to achieve intent, or successful efforts to realize alternative statements of intent that are not inconsistent with the initial statement, but more likely to achieve beneficial results under changed circumstances.
Park & Park (2019)	Based on Pulakos et al.’s (2000) work, they define adaptive performance as flexible work behaviours that help employees adapt to change by demonstrating excellence in problem solving, uncertainty/stress/crisis control, new learning, and adaptability related to people, culture and environment.
Foster, C.J., Plant, K.L. & Stanton, N.A. (2019)	Adaptation encompasses the ability of complex systems to self-organize, reconcile conflicting goals, re-evaluate priorities, and innovate and cope with new external demands. It also refers to the tacit acceptance of broken rules and stretching boundaries to achieve safety performance. The grounded theory approach identified that adaptation is work conducted in a context that is unpredictable, and that involves an explicit or implicit trade-off against conflicting goals. It is a decision, conscious or unconscious, to violate a rule or procedure, or to improvise and work-around a deficiency in the system. Adaptation requires a set of skills and competencies to support the decision-making processes that build upon previous knowledge and experience.
Wallin, A., Nokelainen, P. & Mikkonen, S. (2019)	It refers to a professional’s personal efforts aimed at deliberately improving his or her professional competence, seeking alternative solutions for existing professional practices and becoming an active knowledge-building and networking actor in his or her professional field in order to reach the highest levels of professional competence.
Stasielowicz, L. (2020)	Performance adaptation is used as an umbrella term throughout the article for all similar terms: adaptive performance, adaptability, adaptive transfer, post-change performance. Dealing with change is crucial to performance adaptation.
Kua, J., Lim, W.S., Teo, W. & Edwards, R.A. (2021)	Those with adaptive expertise are not only capable of solving common problems, but also possess a deeper understanding about why the solutions work. They possess conceptual knowledge about ‘why’ things work in addition to procedural knowledge. They demonstrate analogical problem solving and are creative, transferring skills to unfamiliar situations resulting in the ability to balance innovation with efficiency when faced with novel, unusual problems. Adaptive expertise extends beyond critical thinking. Critical thinking refers to the cognitive process that is used to analyse knowledge. It is the adoption of strategies that allows for this transfer of knowledge to different contexts that completes the definition of being an adaptive expert.

One of the main agreements that immediately catches the eye in Table 3 is that adaptive expertise has to do with changing tasks or environments and getting accustomed to this change, which follows the initial conceptualization of Hatano & Inagaki in 1986.

However, as Bohle Carbonell et al. mentioned in 2014, there is no clear description as to how large the change in task or environment must be in order to require an individual to possess adaptive expertise or demonstrate adaptive performance. In our research, we found examples of changes that are new to the entire world, such as the sudden closure of US airspace following the 9/11 terrorist attacks in the US (Foster et al., 2019). This change requires new behaviour from everyone that is confronted with it. Most often this change is characterized as radical and/or societal. We also found examples of changes that are not new to the entire world, but do ask adaptive expertise of a professional or student who first encounters a certain change, such as the adaptive expertise needed in an organizational change context (Park & Park, 2019) or being confronted with new learning tasks (Bohle Carbonell et al., 2014). This kind of change calls for new behaviour that is new specifically to the professional or student involved.

### Conceptual models

To further unravel the concept, we distilled models and frameworks from the selected articles. Comprehensive analysis of the models and frameworks is available (see: Availability of data and materials). To answer the sub question of our first research question we analysed two models that further mapped the concept. Both models have the concept of change in view, which is in line with the conceptualizations in Table 3.

The model of Baard et al. (2013) explains what kind of change is involved in two different ways. It represents levels at which task complexity can vary (component, coordinative and dynamic) and it represents the way in which change is expressed (cognitive, affective/motivational or behavioural). The model of Ward et al. (2018) is about the processes that are put into motion when adaptation is requested. Due to a change in task or environment, data must be reframed and goals need to be adjusted.

### Characteristics related to adaptive expertise

Table 4 depicts characteristics that relate to adaptive expertise.

**Table 4:** Characteristics related to adaptive expertise or adaptive performance

Individual characteristics	(Job) task and/or training characteristics	Characteristics of the learning or work environment
• General cognitive abilities and skills ^(1, 2, 4, 5, 8)^ • Intelligence ^(3)^ • Cognitive flexibility ^(4, 9)^ • Domain specific knowledge and skills ^(1, 5, 6)^ • Prior adaptive experience ^(2, 5)^ • General adaptability ^(3)^ • Role structure adaptation ^(2)^ • Problem solving skills for integrating different components of expert knowledge ^(7)^ • Connecting, relating and synthesizing knowledge ^(9)^ • Deep understanding of problems, seeing problems as an ongoing, iterative process ^(7, 9)^ • Achievement motivation and orientation ^(1, 2)^ • High intrinsic motivation ^(9)^ • Openess to novelty, experience and multiple perspectives/ embracing complexity ^(2, 3, 9)^ • Conscientiousness ^(2)^ • Extraversion ^(2)^ • Neuroticism/ emotional stability ^(2, 3)^ • Agreeableness ^(2)^ • Mental health ^(3)^ • Job experience, satisfaction and performance ^(2)^ • Mastery/learning orientation (in contrast to performance orientation) ^(2, 5)^ • Self-regulatory mechanisms ^(1, 2, 5)^ • Self-efficacy ^(2, 5)^ • Control ^(3)^ • Optimism and positive mindset towards learning ^(3, 9)^ • Coping strategy, hardiness ^(3)^ • Critical thinking ^(4)^ • Sensemaking/adaptive framing ^(4)^ • Flexible execution/flexecution ^(4)^ • Improvisations/ creativity ^(6)^ • Innovativeness ^(9)^ • Collaborative ^(9)^ • Clear communication ^(9)^ • Reflective ^(9)^ • Political skills ^(1)^	• Solving ill-defined or non-routine problems ^(7)^ • Complexity preservation / task difficulty / using abstract materials ^(1,4, 8, 9)^ • Variability of cases ^(4, 9)^ • Task interdependence ^(1)^ • Case-proficiency scaling ^(4)^ • Decision-making autonomy/ goal choice ^(5)^ • Predictability of consequences ^(6)^ • Deliberate practice ^(7)^ • Conceptual tools to master complicated ideas, learning tasks and theoretical work (linked to authentic and practical work situations) ^(7)^ • Facilitating integration of conceptual ideas with existing knowledge ^(9)^ • Guided discovery with hypothesis testing and problem solving ^(9)^ • Metacognitive and challenge-based instruction ^(9)^ • Instructional methods in which students are motivated to explore and discover ^(1)^ • Learning from errors (error cases) or difficult, conflicting or paradoxical situations ^(5,7)^ • Reflection triggered and inquired by experiences, learning and errors ^(4, 7, 9)^ • Problem solving in interaction with others, collaborative learning or social learning, interdependent knowledge-wise ^(7)^	• Supervisor behaviour in creating a supportive work environment in which employees feel valued ^(1)^ • Team learning climate, sharing of knowledge: creating and facilitating connections ^(2, 5, 6, 7)^ • Conductive and safe learning environment for collaborative discourses ^(9)^ • Learning organization, supporting learners’ development ^(5, 7)^ • Mentoring guided feedback and guidance ‘at an arm’s length’ ^(7, 9)^ • Encouragement and authority to explore ^(4)^ • Being confronted with novel situations and learning new tasks ^(1, 4)^ • Prior knowledge and experience as starting point ^(7)^ • Constant curricular review as reinforming factor ^(9)^ • Team structure manipulations ^(2)^ • Training inductions (goal manipulation) ^(2)^ • Communication and assisting processes ^(2)^ • Transformational leadership ^(5)^ • Clear vision with a climate for innovation ^(5)^ • Boundary crossing to expand perspectives ^(7)^ • Negative influence of job uncertainty on adaptive performance ^(5)^

The characteristics represented in Table 4 can either be individual characteristics (column 1), (job) task and/or training characteristics (column 2) or characteristics of the learning or work environment (column 3). This triad is based on the triad used by Bohle Carbonell et al., (2014), where the categories were made slightly broader to categorize elements of the HRD context. Only the characteristics for which at least one author provides evidence of relevance to adaptive expertise were adopted. Specifically, this means that the original source (the review) states that there is evidence for this relation, either based on empirical studies, or based on traceable theoretical reasoning and relations. All the information about the characteristics per review can be found in the additional material (see: Availability of data and materials).

An analysis of the individual characteristics shows that most authors assume domain-specific and more conceptual, declarative knowledge to be one of the main characteristics related to adaptive expertise. Besides knowledge, a few other commonly mentioned skills and attitudes are found. A problem that arises when analysing these characteristics is that there is no complete overview or consensus on the nature of the characteristics. It is unclear whether the characteristics mentioned are more on the output side, being adaptive performance (following the notion that adaptive performance is the visible behaviour resulting from adaptive expertise) or on the input side (requirements or characteristics of a person that lead to adaptive expertise).

The characteristics related to (job) tasks or training and the learning or work environment provide an insight into curriculum development or HRD strategies in order for students or professionals to obtain adaptive expertise. It is found to be important that learners are confronted with a variety of ill-structured and non-routine (real life) tasks, and that they are stimulated to learn and discover by themselves. Learners should be confronted with complex tasks that necessitate problem solving in interaction with others. This is in line with the concept of ‘change’ that we have seen in the conceptualization. Apparently, we ask learners and professionals to show how they deal with change by confronting them with ill-structured or non-routine problems.

Common important characteristics of the learning or work environment are supportive supervisor behaviour, support from co-workers, and a team learning climate allowing learning and innovation. This implies that learners are allowed to make mistakes and that feedback based on these mistakes is crucial to learn from this.

The listed characteristics of the individual, the task or training, and the environment, are a summary of what we have found in the literature. In the discussion section, we will expand on it with respect to what this brings us.

**Fig. 3:**
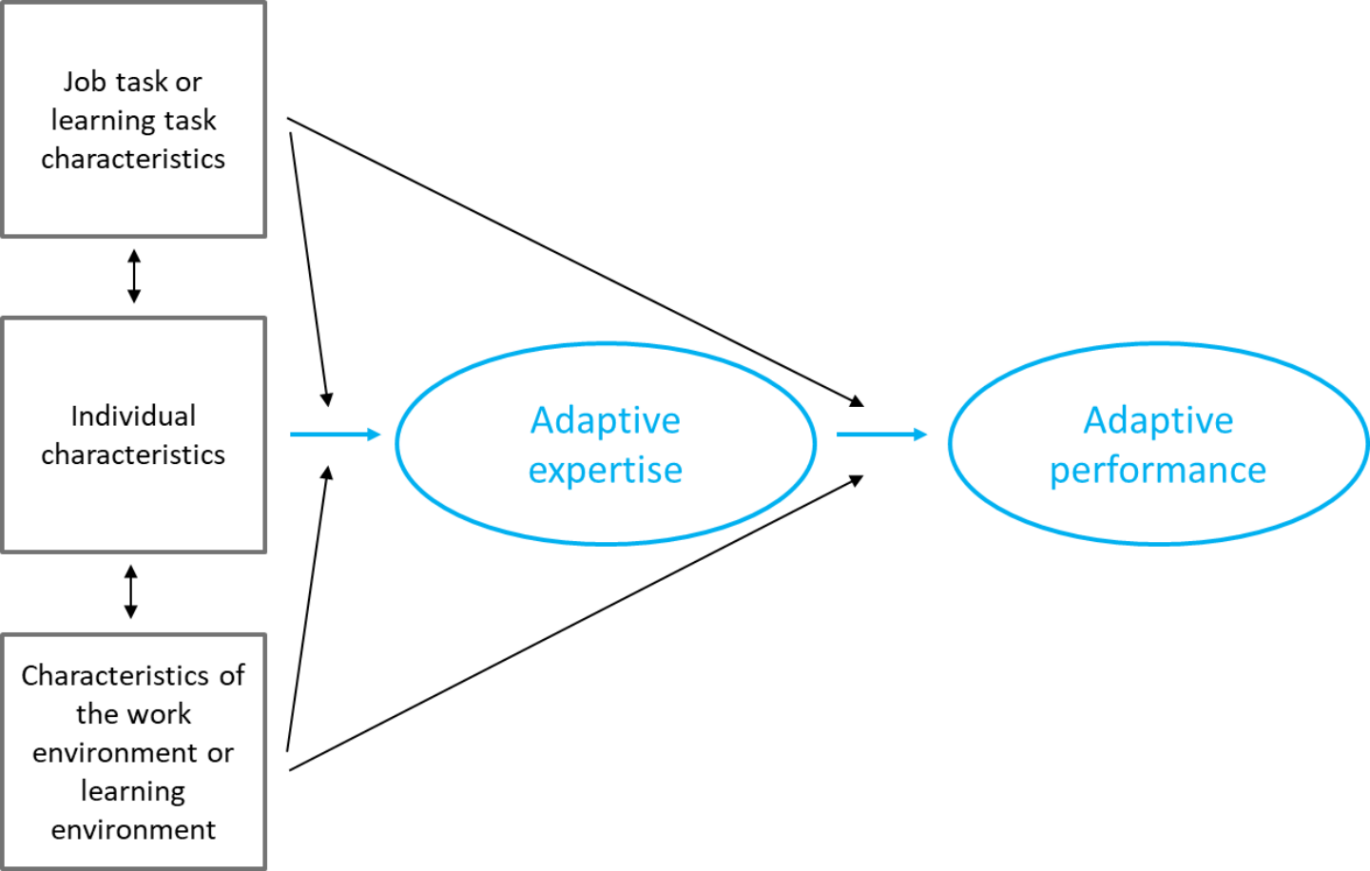
Theoretical representation of the results, including characteristics of the job or learning tasks, individual characteristics and the work or learning environment

Based on all the information we have extracted from the included publications, we can further extend our theoretical representation of the results (Fig. 3). Adaptive performance as a visible expression of adaptive expertise is still the core. As indicated earlier, this is neither a linear relationship nor a causal relationship. Then we added the work- or learning task and the work- or learning environment in the figure. The characteristics of the task or the environment require adaptive expertise and become visible through adaptive performance. For example ill-structured tasks or a climate for innovation (see Table 4). Again, these lines are explicitly not causal relationships, but a representation of a theoretical construct. Also, the individual is added in the figure. This individual possesses different characteristics, for example domain specific knowledge and skills (see Table 4). These characteristics ensure that the individual is able to deal with tasks or an environment that askes for adaptive expertise.

## Discussion

### General discussion and future research

The first aim of our overview of reviews was to contribute to the ongoing scientific debate on the subject of adaptive expertise and adaptive performance by providing more insight into the conceptualization of these concepts, leading to increased conceptual clarity.

Our results further unravel the conceptualization of adaptive expertise and adaptive performance. Although not used unambiguously by all authors, adaptive performance is best referred to as the visible expression of adaptive expertise. As the operationalizations in the result section show, this visible expression is triggered by a ‘changing task or environment’, independent of whether these changes encompass new situations for an individual or a new situation for the whole world. Learners and professionals are tempted, and in many cases forced, to find new solutions in reaction to change, for example in working on ill-structured or non-routine problems. In order to do so, they need adaptive expertise.

The model of Baard et al. (2013) defines elements of change. In addition, Bednall and Henricks (2021) elaborate in their book chapter on the prominent change management theories that have provided important insight into how to lead change effectively and relate that to adaptive performance. They state that traditionally ‘change’ is characterized as episodic, with a clear beginning, middle and end. Bednall and Henricks (2021) indicate that change in relation to adaptive performance could also be ongoing. A context of continuous change may require adoption of new roles or responsibilities, abandonment of important past accountabilities for example. What this explanation makes clear is that the ‘change’ found in our research can have multiple manifestations. As we indicated earlier, it can be either new to the world or new to the learner. Possibly there are other elements that define the type of change. Further research is needed to investigate the appearances of ‘change’.

The second aim of our overview of reviews was to elaborate on what characteristics relate to adaptive expertise and adaptive performance. We have been able to sum up numerous characteristics of an individual, (job) task or work/learning environment that have been shown to be related to adaptive expertise. As indicated before in this paper, the characteristics add up to a long list. Analysing these characteristics leads to several conclusions and recommendations which we will discuss below.

Firstly, cognitive ability or domain-specific knowledge is an individual characteristic mentioned several times by different authors. What remains unknown is what degree of cognitive ability is needed in relation to the degree to which a task is ill-structured or novel. And what kind of environment is needed. It is likely that this is different for a learner, a novice or a professional. This brings us to the point that there is a gap in the existing literature in terms of a good elaboration of the *relation* between the individual, (job) task and environment characteristics, and how these facilitate the development of adaptive expertise and adaptive performance. Several authors have published lists of what is needed to develop adaptive expertise (e.g. Hatano and Inagaki, 1986 and Myloupulos, 2018b). However, as the development of adaptive expertise and adaptive performance are long-term continuous processes in which the separate characteristics all are contributing factors, development can only be optimized when attention is paid to all the characteristics. It is a dynamic system. For example, following the characteristics mentioned by Bohle Carbonell (2014) et al., professionals with very broad specific knowledge and good regulative processes might need less supervisor behaviour in creating a supportive work environment than students or learners with less knowledge and only basal regulative processes. Further research on long-term development that goes beyond identifying sole characteristics and instead focusses on the relation between the characteristics and the different needs of learners, novices and experienced professionals might constitute a huge step forward.

Secondly, as mentioned before, there is no complete overview or consensus in the literature on the nature of the individual, (job) task and environmental characteristics in relation to adaptive expertise and adaptive performance. We often do not know whether the characteristics mentioned are more ‘input’ related and thus related to adaptive expertise or more ‘output’ related and thus related to adaptive performance. An example is ‘self-regulation’. On the one hand this concept is a catalyst for adaptive expertise. On the other hand, it can be visible in the behaviour of a professional that shows adaptive performance. This double position complicates curriculum development that aims to stimulate the development of (future) professionals’ adaptive expertise: should curricula or HRD strategies focus on encouraging self-regulation, or should self-regulation be used as an outcome to measure adaptive performance?

Thirdly, we expect that a number of the (job) task and environmental characteristics found are not specific to the development of adaptive expertise or performance, and are instead merely generally accepted characteristics of good curricula and good HRD strategies. An example of this is supervisor behaviour: in order to develop adaptive expertise or adaptive performance supervisors should be responsive, able to create a supportive work environment, encourage and authorize learners to explore and show transformational leadership. However, in our view, in order to avoid long lists of characteristics, these should be specified for the development of adaptive expertise and adaptive performance, and should be linked to more specific competencies of the supervisor. An interesting question that arises here is whether a supervisor should be an adaptive expert themselves in order to stimulate the development of adaptive expertise and adaptive performance.

Lastly, an analysis of the characteristics did not make it clear whether changes (in task or environment) that are new for the entire world and changes that are new for specific learners call for different individual characteristics. This notion is important because it has consequences for the characteristics that are deemed to be related to the development of adaptive expertise or adaptive performance. Are these characteristics different for changes that are new for the entire world, or are these characteristics the same for everyone who encounters change? Knowing this would inform us about possible ways to train for adaptive expertise to learners and professionals at different stages of their education or career, and in response to different kinds of change.

With Figs. 2 and 3 we made a first attempt to further conceptualize adaptive expertise and adaptive performance and to integrate the job or learning tasks, the work or learning environment and individual characteristics. Even though this is, in our opinion, a good step forward, further operationalization is needed. We presume that operationalization is needed for measuring adaptive expertise or adaptive performance. This would provide further insight into conceptualization, for example on input and output related characteristics. Bohle Carbonell et al. (2014) already recognized this, but added that measuring adaptive expertise is difficult because clearly defining the characteristics is conditional. In 2021, Kua et al. added that there is a dearth of validated measurement tools, partly because the construct of adaptive expertise is defined by the domain of expertise and not easily generalized to other contexts. In our search for review articles, we came across several additional publications on measuring adaptive expertise and adaptive performance. We expect that a thorough overview of the measures, scales and items employed might provide a deeper and broader insight into how different authors operationalize and thereby conceptualize adaptive expertise and adaptive performance.

Our last recommendation relates to workplace learning. In order to be confronted with change, learning tasks or work-related tasks have to be ill-structured, complex, authentic, and either be new to the individual or new to the world. Learners or professionals need ‘space to discover’. These requirements are optimal in (simulated) work environments where either learners or professionals have guided possibilities to work on tasks that call for adaptive expertise and adaptive performance. Workplace learning provides this authentic learning environment, and we suggest further research into the relation between workplace learning and the development of adaptive expertise and adaptive performance.

### Limitations and strengths of the research

Our overview of reviews faces some limitations. One limitation is the exclusive focus on review articles. Review studies are a good source for conceptualization, but there may also be very usable original studies on conceptual interpretation, which we now did not take into regard in our data. Furthermore, we limited our search to publications from between 1986 and 2021, and published in English, German or Dutch. Next, we used keywords in our search related to individuals (Table 1, feature 1). This omits the development of adaptive expertise and adaptive performance at the group level. And finally, even though we used two very comprehensive databases, it is possible that we missed relevant publications that are only available through other databases.

One of the strengths of our overview of reviews is the thorough search, and the search strategy with an extensive number of keywords divided into different features. The whole process from the initial idea and the formulation of the research questions to the final synthesizing of the results has been carried out by an experienced and multidisciplinary team as described in the positionality paragraph. In addition, we brought together the worlds of expertise research in educational settings and in HRD research and clarified relationships, where previous research used concepts interchangeably because definitions were alike.

## Conclusion

This overview of reviews has provided insight into the different review studies on adaptive expertise and adaptive performance. Although these terms are not used unambiguously by different researchers in the field, the conceptualization of adaptive expertise and adaptive performance, and the relation between these terms has been further unravelled. Adaptive performance is best referred to as the visible expression of an adaptive expert and this expression is triggered by a ‘changing task or environment’. The scope of this ‘changing task or environment’ lies somewhere between ‘new for the learner’ and ‘new for the whole world’.

We found numerous individual, (job) task and environmental characteristics related to adaptive expertise and adaptive performance. The nature and relation of these characteristics, and their specificity in relation to adaptive expertise and adaptive performance are visualized in a coherent figure, but also provide several suggestions for future research.

## Data Availability

All additional material is available online (www.adaptatwork.nl/en) and upon a reasonable request at the corresponding author.
